# Accounting for Nature's Benefits: The Dollar Value of Ecosystem Services

**DOI:** 10.1289/ehp.120-a152

**Published:** 2012-04-01

**Authors:** David C. Holzman

**Affiliations:** **David C. Holzman** writes on science, medicine, energy, economics, and cars from Lexington and Wellfleet, MA. His work has appeared in *Smithsonian*, *The Atlantic Monthly*, and the *Journal of the National Cancer Institute*.


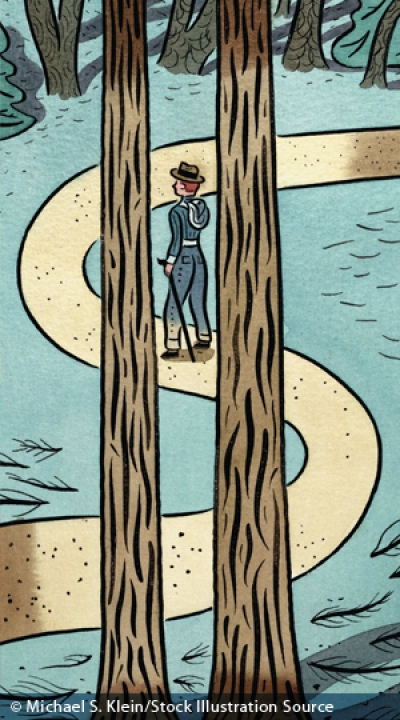
Healthy ecosystems provide us with fertile soil, clean water, timber, and food. They reduce the spread of diseases. They protect against flooding. Worldwide, they regulate atmospheric concentrations of oxygen and carbon dioxide. They moderate climate. Without these and other “ecosystem services,” we’d all perish.^1^

One hallmark of the history of civilization is an ever-increasing exploitation of ecosystem services coupled with substitution of technology for these services, particularly where ecosystems have been exploited beyond their ability to provide.^2^ Agriculture is a hybrid of exploitation and substitution that enabled people to live in greater, denser populations that drove further exploitation and substitution. Modern plumbing made close quarters far less noxious but led to exploitation of ecosystems’ ability to break down sewage, and to substitution with expensive sewage treatment technologies. Exploitation of fossil fuels led to a slew of modern conveniences, including fishing fleets that are so effective at catching their prey that they threaten fisheries globally.^3,4^ All this exploitation strained ecosystems, but in the past, when the population was a fraction of what it is now, these strains were local rather than global phenomena.

In 2005 the Millennium Ecosystem Assessment (MA),^5^ a sweeping survey conducted under the auspices of the United Nations, found that approximately 60% of 24 ecosystem services examined were being degraded or used unsustainably.^6^ “Every year we lose three to five trillion dollars’ worth of natural capital, roughly equivalent to the amount of money we lost in the financial crisis of 2008–2009,” says Dolf de Groot, leader of the Research Program on Integrated Ecosystem Assessment and Management at Wageningen University, the Netherlands.

The value of ecosystem services typically goes unaccounted for in business and policy decisions and in market prices. For commercial purposes, if ecosystem services are recognized at all, they are perceived as free goods, like clean air and water. So it’s not surprising that much of the degradation of ecosystems is rooted in what the President’s Council of Advisors on Science and Technology (PCAST), an independent group of U.S. scientists and engineers, describes as “widespread under-appreciation of the importance of environmental capital for human well-being and . . . the absence of the value of its services from the economic balance sheets of producers and consumers.”^7^ PCAST and other groups are working to build recognition of ecosystem services and, importantly, to valuate them—that is, calculate values for these services to help policy makers and resource managers make rational decisions that factor important environmental and human health outcomes into the bottom line.

## An Idea Whose Time Has Come?

In July 2011 PCAST called upon the federal government to assess quadrennially the condition of the nation’s ecosystems and the social and economic value of services they provide. The goal was to improve methods for evaluating those services and to establish an ecoinformatics initiative to pull together existing knowledge and gather new information in a format that interested parties can easily use. ^7^

But the concept of valuating ecosystem services is not new. John P. Holdren, now science advisor to President Barack Obama, introduced it to students in his class “Quantitative Aspects of Global Environmental Problems” at the University of California, Berkeley, in the 1970s. He emphasized that technological substitutions for ecosystem services are often costly, sometimes to the point of impracticality, and that sometimes an incomeplete understanding of how they function makes such substitutions impossible. Geoengineering to mitigate global climate disruption in the face of increasing emissions, for example, is widely viewed as extremely risky, because the climate is so complex.

In 1997 Robert Costanza, Distinguished University Professor of sustainability at Portland State University, Oregon, and colleagues first estimated that ecosystem services worldwide are worth an average $33 trillion annually ($44 trillion in today’s dollars), nearly twice the global GNP of around $18 trillion ($24 trillion in today’s dollars).^8^ Although the $33 trillion has been difficult to substantiate, this study was widely praised for drawing attention to the value of ecosystem services, says Rick Linthurst, national program director of the Ecosystem Services Research Program at the U.S. Environmental Protection Agency (EPA).

Payments to preserve ecosystem services date to at least the early 1980s, when the United States implemented wetland and stream credit banking,^9^ but the idea really took off in the mid 1990s. For instance, in 1996 Costa Rica began paying landowners $42 per hectare per year to preserve forest.^10^ At the time, that country had the highest deforestation rate in the world; now it has among the lowest, says Gretchen C. Daily, Bing Professor of Environmental Science at Stanford University.

China responded to a devastating drought in 1997, followed by massive floods in 1998, by inaugurating various payments for ecosystem services and a policy for conserving areas that are important sources of ecosystem services. These are known as Ecosystem Function Conservation Areas. Among the benefits: soil erosion fell sufficiently to cut sediment in the Yellow River by 38% over the period 2000 through 2007, and carbon sequestration rose by an estimated 1.3 billion tons between 1998 and 2010, says Jianguo Liu, the Rachel Carson Chair in Sustainability and University Distinguished Professor of fisheries and wildlife at Michigan State University. But he adds that some benefits probably came at the expense of natural capital elsewhere in the world, as declines in forest cutting coincided with a rise in imported timber.

In 2010 the World Bank launched a program to help countries incorporate the value of ecosystem services into their accounting systems with an eye toward managing ecosystems to maximize economic benefit.^11^ Colombia, beset for several years by unusually persistent and damaging rains, is one of five pilot countries working with the bank.

Elsewhere, Norway is paying Indonesia $1 billion to preserve rainforest for carbon storage and sequestration to limit the impacts of climate change.^12^ And in Vietnam, an investment of $1.1 million in mangroves, which protect coastal regions from flooding, saved $7.3 million annually that would have gone to maintaining dikes. ^1^

A number of agencies of the U.S. federal government are conducting research on ecosystem services. The EPA is assembling a national atlas that can overlay visual information, like that used in Google Earth, with ecological and economic analyses to reveal variability in ecosystem service provision. The agency also has pilot programs in four regions of the United States enabling interested parties to project different resource-use scenarios into the future to help guide decision making now.^13^

And in 2007 environment ministers from the G8+5 countries^14^ agreed to begin analyzing the global economic benefits that derive from ecosystems and biodiversity, and to compare the costs of failure to protect these resources with the costs of conserving them. In the ensuing years, the resulting initiative, The Economics of Ecosystems and Biodiversity (TEEB),^15^ has produced a series of reports for decision makers at the international, national and local levels aimed at enabling practical responses.

Another leader in guiding decision makers on payments for ecosystem services is the Natural Capital (NatCap) Project, cofounded by Stanford’s Daily in 2006.^16^ NatCap has created software called InVEST (Integrated Valuation of Environmental Services and Tradeoffs) to model tradeoffs among environmental, economic, and social benefits, so that decision makers can explore the implications of alternative land-use scenarios. For any given piece of real estate, InVEST can take existing data on various ecosystem services—each of which may fall under a different field of study—and provide one consistent platform for assessing all of them together, to determine the optimal use(s) of that land, says Heather Tallis, lead scientist of NatCap. For example, “trade-off curves” can reveal how much timber can be harvested before causing major profit loss to hydropower, flood damage, or loss of biodiversity. The tools are available free through NatCap.^17^

**What Are Ecosystem Services?**

Ecosystem services are highly interdependent and often overlap. These services are typically categorized under 4 types: provisioning, regulatory, supporting, and cultural.^21^

Like factories, **provisioning services** maintain the supply of natural products: food, timber, fuel, fibers for textiles, water, soil, medicinal plants, and more.

**Regulatory services** keep different elements of the natural world running smoothly. They filter pollutants to maintain air and water quality, moderate the climate, sequester and store carbon, recycle waste and dead organic matter, and serve as natural controls for agricultural pests and disease vectors.

**Supporting services** can be thought of as the services that maintain the provisioning and regulatory services. These services include soil formation, photosynthesis, and provision of habitat. Healthy habitats preserve both species diversity and genetic diversity, which are critical underpinnings of all provisioning and regulatory services.^22^

Finally, **cultural services** are defined as the intangible benefits obtained from contact with nature—the aesthetic, spiritual, and psychological benefits that accrue from culturally important or recreational activities such as hiking, bird watching, fishing, hunting, rafting, gardening, and even scenic road trips. Increasingly, these services are being tied to tangible health benefits, especially those related to stress reduction.^23^

NatCap’s consulting group is currently working on numerous projects within the United States and with 15 other countries in Africa, Latin America, the Pacific, North America, and Asia. Foremost among those countries is China, which is spending a total of around $100 billion—more than any other country—to preserve forestlands through logging bans, to buy farms that are perched unsustainably on steep slopes for conversion to forests, and to restore wetlands.^18^ The Chinese government will shift farmers either to more sustainable locations or to other occupations, says Daily. NatCap is using InVEST to assess how many resource-intensive livelihoods—in farming, forestry, herding, and other fields—could be supported sustainably in a certain area under given practices, and to evaluate how shifting inhabitants to an alternative mix of livelihoods would impact natural capital and ecosystem services. This helps inform the investments needed to enable desired shifts, as well as to ascertain who will benefit and who will be hurt by the shifts, and to determine appropriate compensation, says Daily.

## Assigning a Dollar Value

Ecosystem services are valued, ideally, by how much human welfare they can provide. The most convenient measure of welfare is dollars, although at this early stage of development of the science, that is not always a practical measure.

Values for provisioning services [see sidebar, “What Are Ecosystem Services?”] are relatively easy to determine. The simplest and least controversial methods to assess value draw on existing prices in the marketplace, says Emily McKenzie, manager of the NatCap Project at the World Wildlife Fund U.S. office. For example, coastal and marine ecosystems support the production of fish. The value of this service can be assessed based on revenues, a function of the price and quantity of harvested fish.

Thus, the value of the provisioning service is equal to how much all of its current and future production is worth today—what economists call its “present value.” The further into the future the production lies, the lower the present value of the service. That’s because money invested today in a safe investment, such as a Treasury bill, almost certainly will grow. If Treasury bills are earning 3%, $100 invested today will become $103 a year from now, $106.09 two years from now, and so on. That means that $106.09 two years from now is no more valuable than $100 today.

Many ecosystem services, such as scenery, recreational value, and most regulatory services, including those moderating infectious disease, lack a market price. One way to address this problem involves asking people what they would pay for a particular service, says Stephen Polasky, Fesler-Lampert Professor of ecological/environmental economics at the University of Minnesota; this is called “stated preference.” Another method, “revealed preference,” involves determining values from related actual purchases, such as the money people spend to travel to bucolic tourist destinations, or the extra cost of a house with a water view over a similar nearby house without the view.

Another valuation technique is estimating “replacement cost.” This is the cost of the least expensive technical fix as a replacement for an ecosystem service. For example, New York City recently paid landowners in its watershed more than $1 billion to change their farm management practices to prevent animal waste and fertilizer from washing into the waterways. In doing so, the city avoided spending $6–8 billion on a new water filtration plant and $300–500 million annually to run it—the replacement cost of the natural filtration provided by waterways.^1^ “Protecting the watershed [along with the ecosystem service it provides] can be said to be worth at least six to eight billion dollars because that is the cost of replacing the service,” says Polasky, who notes that the value of clean water is far higher still.

**Figure:**
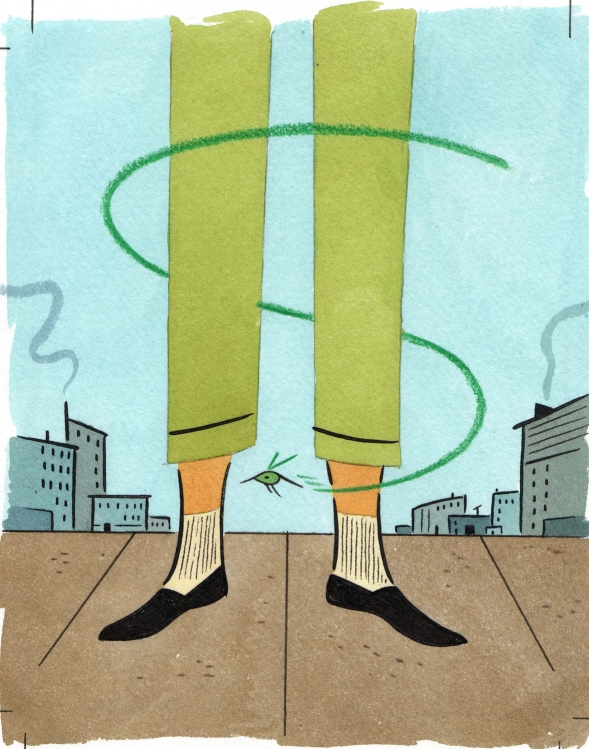
**Quantifying the Impact of Ecosystem Disturbances on Infectious Disease** The idea that ecosystem services influence human health has been around for quite a while, says Rick Ostfeld, a disease ecologist at the Cary Institute of Ecosystem Studies in Millbrook, New York. Only more recently have researchers begun investigating the hypothesis that services provided by healthy ecosystems include moderating infectious disease. There is growing evidence—some experimental and some correlational—that a decline in biodiversity can boost disease transmission and that “preserving intact ecosystems and their endemic biodiversity should generally reduce the prevalence of infectious diseases,” Ostfelt and colleagues wrote in a recent review on the topic in Nature.^24^ When ecosystems are simplified or fragmented, as human development is wont to do, the changes often favor proliferation of more efficient vectors and wildlife reservoirs of infectious disease—chiefly arthropods and rodents, respectively—partly by reducing the population of predators that keep these creatures in check. The most efficient natural reservoirs of disease “tend to be the weedy, resilient species with a ‘live fast and die young’ life history,” says Ostfeld. “Those are the species left standing when we disturb or degrade the ecosystem.” He explains that predators that feed on the natural reservoirs tend to be more sensitive and disappear first when ecosystems are disturbed. There is direct evidence supporting an inverse relationship between biodiversity and infectious disease. In South America, for instance, converting forest to cereal production increases rodent populations, contributing to epidemics of viral hemorrhagic fever, says Samuel Myers, a research scientist in the Department of Environmental Health of the Harvard School of Public Health. Dams, irrigation systems, and deforestation have been linked to increases in malaria and schistosomiasis, diarrheal diseases are associated with road building, and dengue has been tied to urbanization.^25^ Nonetheless, “[t]here is a big gap between the research showing associations between changes in natural systems and health outcomes, and actually being able to quantify the specific health benefits or costs of incremental changes in the system,” Myers and colleague Jonathan Patz of the University of Wisconsin–Madison wrote in the 2009 edition of Annual Review of Environment and Resources.^25^ Illustration by Michael S. Klein

The value of an ecosystem service depends on local and/or regional socioeconomic conditions as well as supply and demand. Thus, the value of clean water is much higher in New York City’s watershed, where it serves 19 million people^7^ than it would be in, say, Alaska, says Polasky.

When it comes to valuating ecosystem services, the economics is the easy part—easy being a relative term. The major difficulties have more to do with the fact that ecology is a relatively young science, and there is much that we don’t yet understand about it, says Polasky, echoing colleagues. “Nature is probably the most complex system we know of,” Daily explains.

## Huge Error Bars and Heroic Assumptions

Part of the problem, generally speaking, is that the multiple uncertainties about how ecosystems do what they do add up to “huge error bars,” says Polasky. Dollar values are often based on “heroic assumptions” that don’t stand on much data, says Lisa Wainger, a research associate professor at the University of Maryland Center for Environmental Science.

**Figure:**
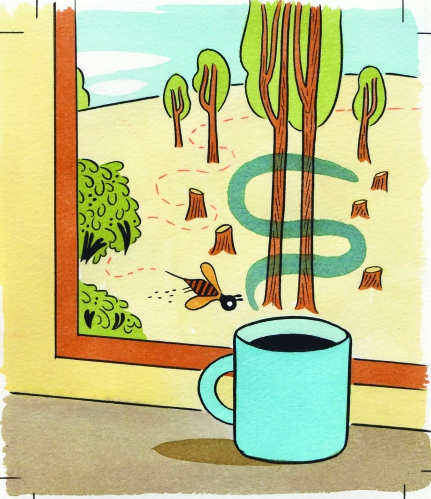
**A Complex Undertaking** The science that underlies ecosystem services is cumbersome. Taylor Ricketts, director of the Gund Institute for Ecological Economics at the University of Vermont, measured the productivity of bees as pollinators of coffee plantations in Costa Rica. The bees live in forests near the plantations. In a series of carefully controlled and lengthy experiments, he found that coffee plants within a kilometer of the forest were fully pollinated, whereas those beyond a kilometer received insufficient pollination, producing 20% fewer coffee beans. Using these results, Ricketts calculated that two forest patches were contributing $62,000 worth of pollination services annually to a single coffee farm. If those forests were destroyed, that farm would suffer a 7% drop in productivity, he estimated. Thus, the present value of the annual $62,000 would be the value of the service provided by those forest patches.^26^ However, pollination is just one of many ecosystem services performed by that forest, with others including carbon sequestration, support of biodiversity, and water purification, to name just a few, says Ricketts. At this early stage of the science of valuating ecosystem services, often it is impractical to determine the value of more than one or a few services. Part of the difficulty of determining the value of all the services within an ecosystem is that the methods for obtaining the necessary information are often so different for each service. In some cases, the studies required may be enormously time-consuming or otherwise difficult. For example, Ricketts’ pollination studies involved comparing coffee production among five similar trees at each of three different distances from the forest, after hand pollinating flowers on some branches at each location to simulate maximum pollination activity, allowing bees to do the job on other flowers, and covering some flowers to simulate no pollination. Measuring a forest’s capacity to purify water might involve determining the purity of a stream that runs through the forest after passing a pollution source by assaying pollutants at regular distances to determine how quickly they are declining. Assaying biodiversity involves taking a census of all plants, animals, and invertebrates on a plot of land. Ricketts says a thorough determination of ecosystem services from a single piece of land might involve 20 different studies for as many services. Illustration by Michael S. Klein

For instance, scientific understanding of feedback among the many ecosystem services remains wanting—“If you have more carbon in soil, are plants better able to take up nitrogen?” asks Polasky, as one example. A good deal of ecological uncertainty stems from a lack of information about basic natural history. The PCAST report notes that “groups of organisms likely to be most important in ecological terms, such as species that determine soil fertility, promote nutrient cycling, or consume wastes . . . are among the least familiar and least visible—e.g., fungi, nematodes, mites, insects, and bacteria. Populations of ecologically dominant marine organisms, most of which are either invertebrates or microbes, are just as poorly understood.”^7^

Climate change magnifies all these ecological uncertainties. It’s “the mother of all externalities,” writes Richard S.J. Tol, a professor of economics at the University of Sussex, “larger, more complex, and more uncertain than any other environmental problem.”^19^ Over the rest of the century, global climate shifts are likely to be the biggest driver of ecosystem change and may greatly reduce Earth’s carrying capacity, according to PCAST.^7^

It also remains difficult to link changes in the delivery of ecosystem services to changes in human welfare. “There are many mysteries about which species confer what dynamics to ecosystems or what benefits to people,” says Daily. “We really don’t know how much biodiversity is needed to sustain and fulfill human life.”

But more precise knowledge of the economic value of those ecosystem services that can easily be valuated “would not, in itself, provide insight into what fraction of the benefit would be lost in consequence of a given type or degree of ecosystem disruption,” according to the PCAST report.^7^ There are thresholds in ecosystem function beyond which carrying capacity plummets. History and prehistory are littered with thresholds breached, from the degeneration of the Fertile Crescent into today’s desertified Middle East (probably due to mismanagement of irrigation, says Daily) to the deforestation, extinction of all wild land birds, and human population collapse on Easter Island.^2^ One of the biggest fears about the impact of climate change is that global thresholds will be breached, but the ability to predict such with anything approaching precision is currently beyond ecological science.

## Progress

Despite the challenges, considerable progress has been made over the last decade toward improved techniques for linking changes in ecosystem services to changes in human welfare. Part of that improvement is due to modeling methods, including InVEST, as well as to greater numbers of ecological studies, and part is due to improvements in the data, says Polasky. The field has been boosted by the revolution in GIS (geographic information system) technology and so-called spatially explicit data: “We now have very good images that enable us to know the heights of plants and elevations of terrain, and really good sensors that show us what’s on the ground,” Polasky says. “You can combine that with monitoring. If we increase the deforestation upriver, we can monitor the sediment downriver. That’s been a huge help.”

Health & Ecosystems: Analysis of Linkages (HEAL),^20^ a consortium of more than 25 conservation and public health institutions, has embarked on the first rigorous, systematic attempt to measure the human health impacts of changes in a variety of natural systems. HEAL’s projects are designed to evaluate what are thought to be key connections between the environment and health. Examples include the relationships between subsistence hunters’ sustainable access to wildlife and their children’s nutritional needs (particularly as related to iron and key micronutrient deficiencies); between upland deforestation on islands such as Fiji, erosion and waterborne diarrheal diseases in children, and downstream coral reef health and productivity; between deforestation patterns and malaria in the Amazon and other major forest systems; between landscape fires in Sumatra and smoke-related cardiopulmonary illness in the broader region downwind; and between fishers’ access to Marine Protected Areas, food security, income to purchase health services, and the psychological dimensions of having a “sense of place” related to coastal resource security.

Perhaps most importantly, says Steve Osofsky, HEAL coordinator and director of health policy at the Wildlife Conservation Society, the project seeks to quantify all these types of relationships related to communicable diseases, noncommunicable diseases, nutrition, and the social and psychological dimensions of health. In Osofsky’s words, “If it cannot be measured, it cannot be managed.”

The ultimate goal of valuating ecosystem services “is to improve human well-being overall,” says Daily. She cautions that there will always be people who lose out in any policy decision. However, she says, “The aim is to design these investments in natural capital so as to advance human development and alleviate poverty at the same time. This is the Holy Grail.”
